# Supplementary material for: Universal emulsion stabilization from the arrested adsorption of rough particles at liquid-liquid interfaces
Source: Nat Commun. 2017 Jun 7;8:15701. doi: 10.1038/ncomms15701 (PMC5467241; doi:10.1038/ncomms15701)
Supplement: Supplementary Information — Supplementary Figures, Supplementary Tables, Supplementary Notes and Supplementary References [file ncomms15701-s1.pdf]

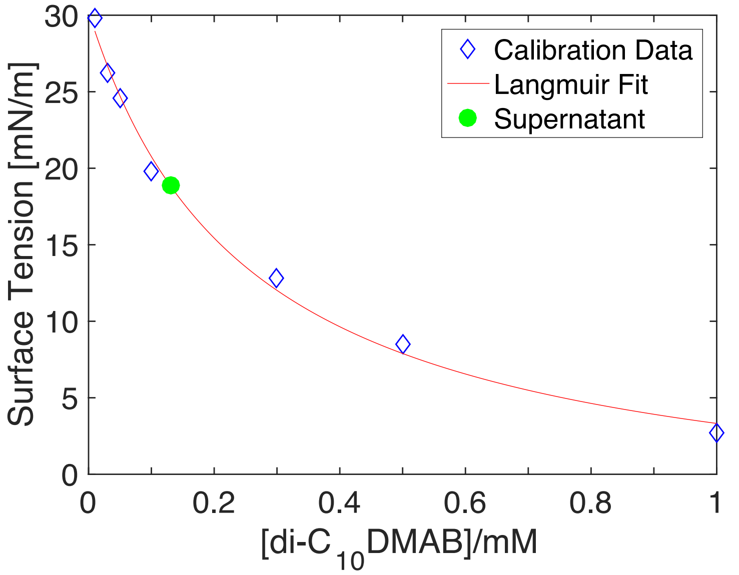

Supplementary Figure 1 Surface tension calibration. Surface tension of aqueous di-C_{10}DMAB solutions against *n*-decane used to calibrate the working conditions of the *in-situ* hydrophobisation of the silica particles prior to FreSCa cryo-SEM. The green circle corresponds to the surfactant concentration used in the experiments.

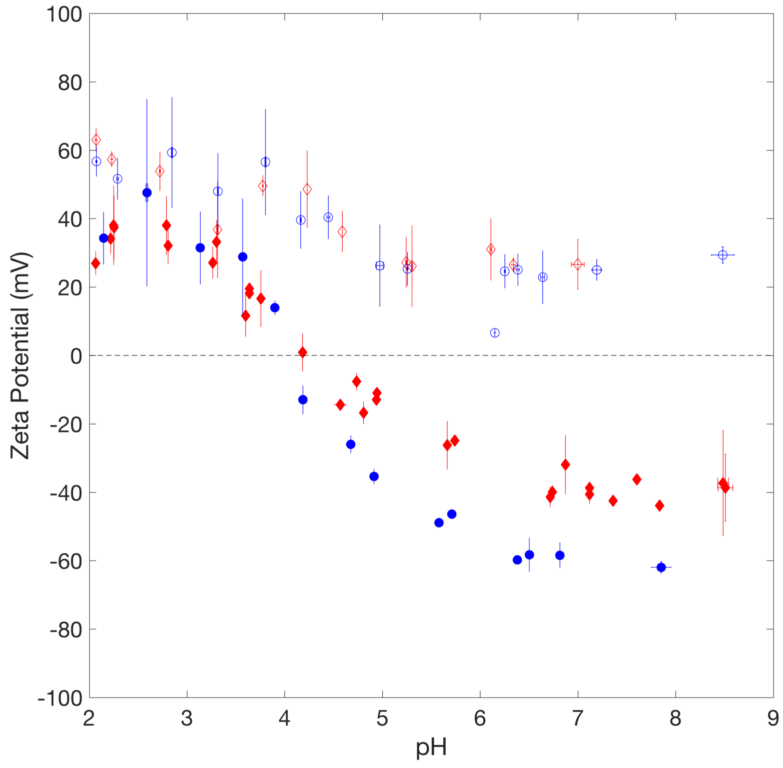

Supplementary Figure 2 Particle electrophoretic mobility. Zeta potential of rough silica particles as function of pH for two smoothening layer thicknesses. Blue and red data points correspond to particles with 60 and 10 nm thick silica layers, respectively (data points IV and VI in Figure 2a of the main text). Filled symbols are for the native particles and the empty ones

correspond to particles in the presence of 0.1 mM of di-C_{10}-DMAB. The zeta potential measurements were repeated 3 times for each selected pH value. Error bars represent the standard deviation of the measured values at each pH point.

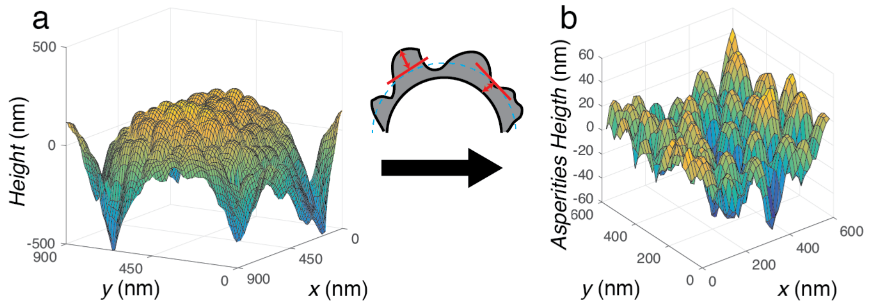

Supplementary Figure 3 Single-particle AFM roughness extraction. The spherical curvature of the particle is removed from the AFM scan **(a)** to obtain a background–free topography signal **(b)**.

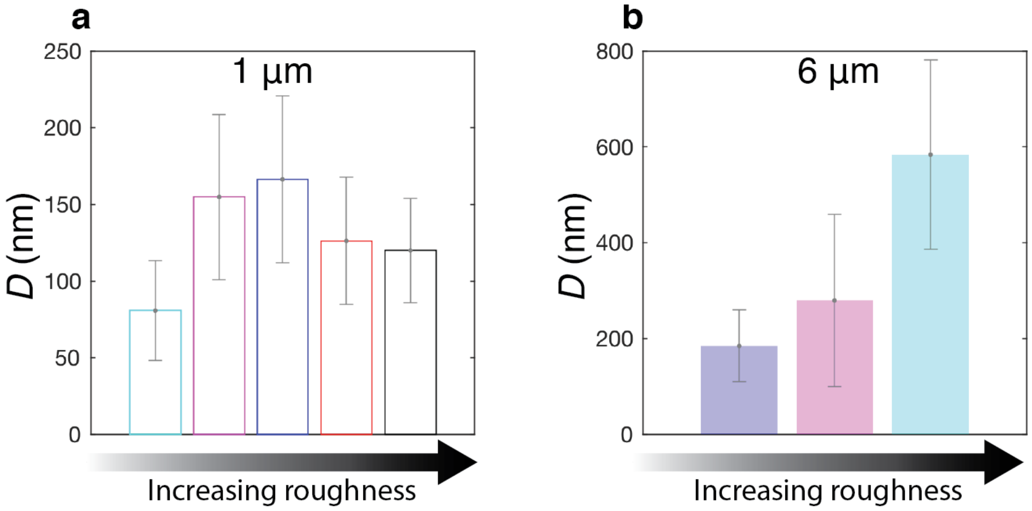

Supplementary Figure 4 Asperity-to-asperity distances. Panel **(a)** and **(b)** show the asperity-to-asperity distance *D* for the produced rough particles with average diameters of 1 μm and 6 μm, respectively. The colours refer to the different roughness classes. Each bar represents the mean value and the error bars indicate the standard deviation extracted from the data distributions, calculated on order 10 particles. In panel **(a)**, cyan corresponds to $RMS = 3.6$ nm, magenta to $RMS = 6.6$ nm, blue to RMS = 7.1 nm, red to $RMS = 12.6$ nm, and black to $RMS = 21$ nm. In panel **(b)**, blue represents particles with $RMS = 17.5$ nm, pink with $RMS = 31.6$ and light blue with $RMS = 54.5$ nm.

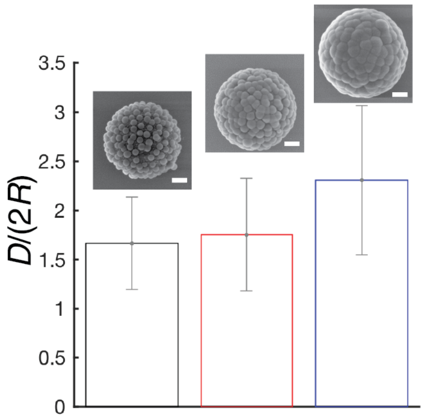

Supplementary Figure 5 Inter-asperity distance after smoothening. The asperity-to-asperity distances D are normalised by the berries' size (radius $R = 36$ nm) for the same family of rough particles. All three batches are produced using the same berries' size. The scale bar in the SEM images is 200 nm. The colours refer to the different roughness classes. The black histogram represents particles with an average *RMS* roughness of 21 nm, red of 12.6 nm and blue of 7.1 nm; see Supplementary Table 1. Each bar represents the mean value and the error bars indicate the standard deviation extracted from the data distributions, calculated on order 10 particles.

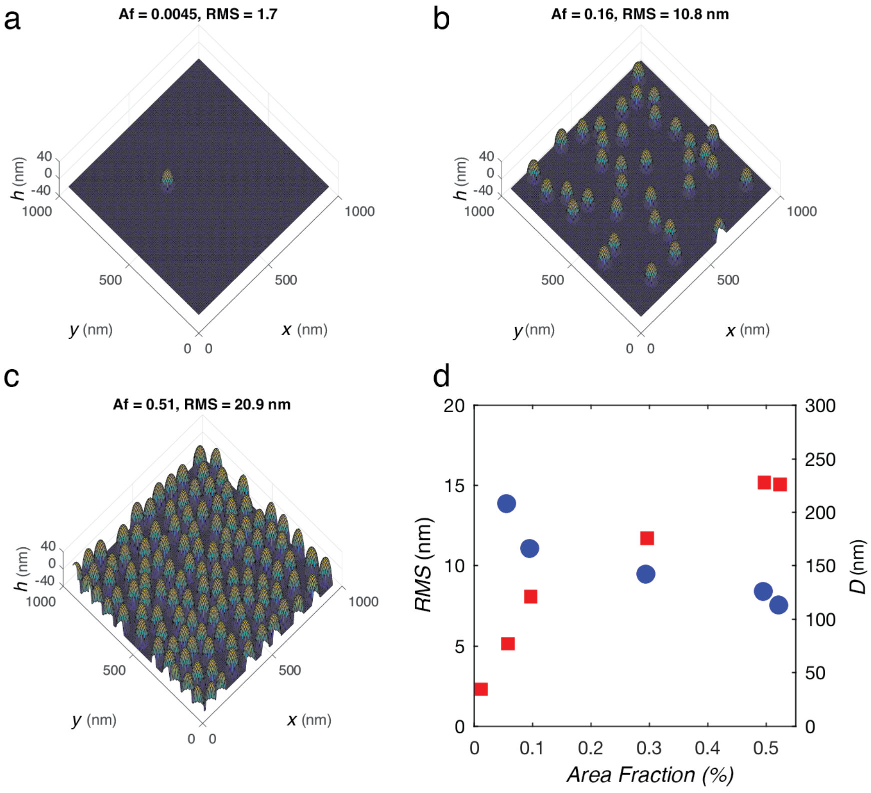

Supplementary Figure 6 Surface roughness simulations. (a) Simulated rough surfaces with spherical asperities of radius R = 36 nm and height $\underline{H}$ = 36 nm. *Af* is the area fraction occupied by the asperities. **(b)** Simulated *RMS* roughness (red squares, left axis) and nearest inter-asperity distance D (blue

circles, right axis) as function of the asperities area coverage. Both the radius (*R*) and height (*H*) of the simulated asperities are 36 nm.

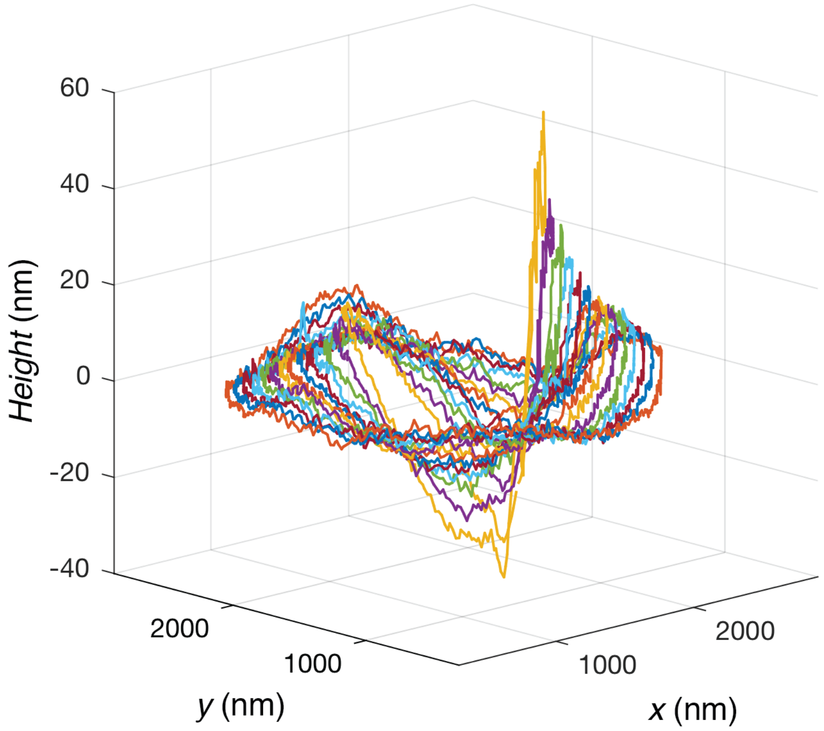

Supplementary Figure 7 Reconstruction of the interface deformations. Discretized three-dimensional reconstruction of the interface profile around a rough particle trapped at a w/o interface. The concentric lines are separated by 50 nm. The particle belongs to the roughness class with an average *RMS* roughness of 12.1 nm. It was functionalised with bromo-silane and it adsorbed spontaneously from n-decane.

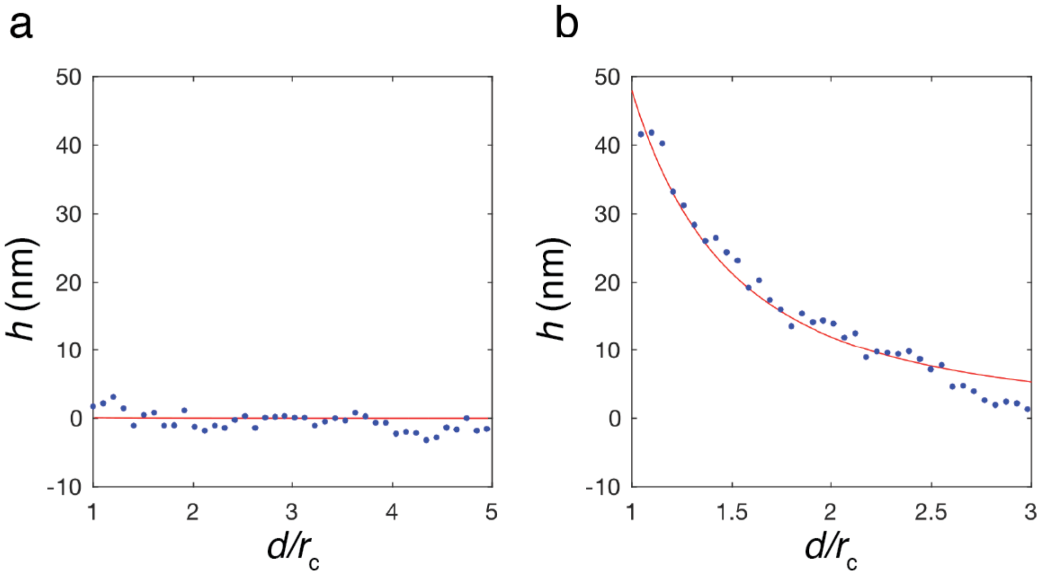

Supplementary Figure 8 Radial profile of interfacial deformation. Interface height versus radial distance d normalized by the cross-sectional radius of the particle at the interface r_c. The blue circles are the experimental data and the solid red lines are fits using the expression in Supplementary

Equation 2. The interfacial deformations are depicted for (a) a smooth OTS-functionalised silica particle and for (b) a rough bromo-silanised particle with *RMS* roughness of 12.1 nm.

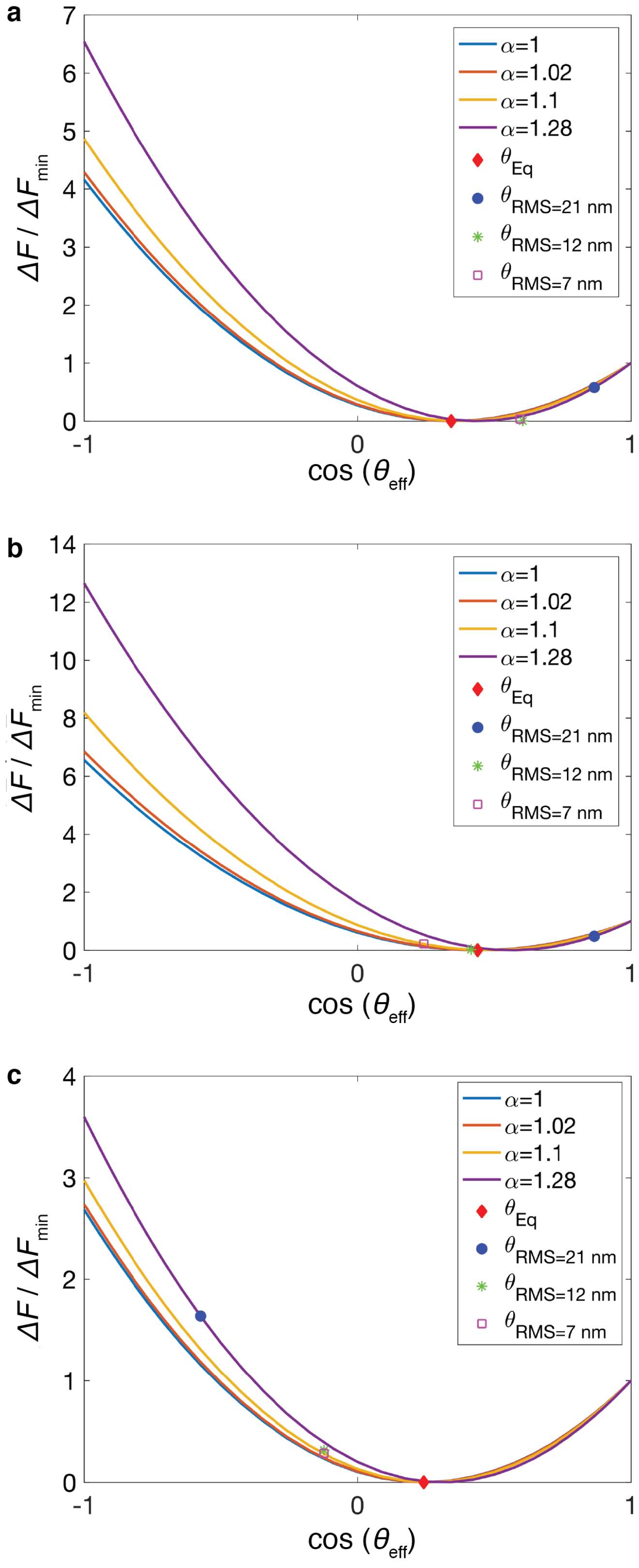

Supplementary Figure 9 Adsorption energy wells. The potential around the equilibrium contact angle is plotted for different surface roughness and

adsorption conditions. **(a)** In-situ hydrophobised rough particles at a w/o interface in the presence of 0.1 mM di-C_{10}-DMAB. **(b-c)** Bromo-silanized rough particles adsorbing from the aqueous phase **(b)** and from the oil phase **(c)**, respectively. α is the measured surface area magnifying factor for the different particle batches. The solid curves are calculated using Supplementary Equation 7. The red diamonds indicate the equilibrium position obtained for smooth and homogeneous particles. The other symbols correspond to the measured values of the effective contact angles measured for the different particle batches.

Supplementary Table 1 Reaction parameters for the fabrication of all-silica raspberry-like (RB) particles. *Refers to 20 mg particles. ** PD refers to a polydisperse sample

Reference main text	RB Type	Berry size (nm)	Injected TEOS (mL) at 5 vol%*	Estimated thickness of the smoothening silica layer (nm)	RMS roughness(nm)
Fig. 2a II	RB_12_1µm	12	0.92	8	3.6
Fig. 2a III	RB_39-12_1µm	39&12	1.46	20	6.6
Fig. 2a IV-Fig. 2b II	RB_72-12_1µm	72&12	2.08	60	7.1
Fig. 2a V-Fig. 2b III	RB_72-12_1µm	72&12	1.04	30	12.6
Fig. 2a VI-Fig. 2b IV	RB_72-12_1µm	72&12	0.34	10	21
Fig. 2c III	RB_72_6µm	72	0.192	10	31.6
Fig. 2c II	RB_72_6µm	72	0.769	40	17.5
Fig. 2c IV	RB_250_6µm	250 PD**	3.852	200	54.5

Supplementary Table 2 Static and dynamic contact angle measurements on flat wafers.

**Wafer modification**	$\theta^{s}_{w/a}$ (°)	$\theta^{r}_{w/a}$ (°)	$\theta^{a}_{w/a}$ (°)	$\theta^{s}_{w/o}$ (°)	$\theta^{r}_{w/o}$ (°)	$\theta^{a}_{w/o}$ (°)
none	24.3 ± 1.4	< 10	26.8 ± 1.4	26.1 ± 0.5	<10	30.2 ± 0.4
α-bromoisobutyryl bromide	69.1 ± 0.3	46.2 ± 1.3	70.3 ± 1.0	100.6± 0.4	80.6 ± 1.3	104.7± 0.9
OTS	107.4 ± 0.8	102.1± 1.2	109.5± 1.5	150.3± 3.2	149.6± 1.1	154.8± 1.7

Supplementary Table 3 Comparison between experimental and simulated roughness data. Only data sets having comparable inter-asperity distances *D* are considered.

Experimental		Simulation			
**RMS (nm)**	**D (nm)**	**R (nm)**	**H (nm)**	**RMS (nm)**	**D (nm)**
3.6	81	12	12	2.8	77
6.6	155	20	25	5	157
7.1	166	36	20	6.5	163
12.6	126	36	25	12.7	117
21	120	36	40	21	121

Supplementary Note 1: Additional details of the particles fabrication

The Supplementary Table 1 describes the synthesis parameters for the fabrication of the raspberry-like (RB) particles. The estimation of the thickness of the smoothening silica layer is based on the full conversion of all the added TEOS over an area corresponding to the total surface area (corrected for the surface roughness) of all the particles in the mixture.

Supplementary Note 2: Contact angle of macroscopic flat substrates

The Supplementary Table 2 summarizes the values of the static and dynamic contact angles measured on flat silicon wafers modified as the rough microparticles shown in Figure 3 of the main text.

Supplementary Note 3: Additional characterization of RB particles before and after in-situ hydrophobisation by cataionic surfactant physisorption

Supplementary Figure 1 displays the calibration of the surfactant concentration for the experimental conditions used in FreSCA cryo-SEM to

measure the contact angle of the RB particles modified by the physisorption of di-C_{10}DMAB.

Supplementary Note 4: Titration curves for raspberry-like particles

Supplementary Figure 2 shows the pH-dependent zeta potential of two rough particles, corresponding to the data points IV and VI of Figure 2a in the main text, respectively, before and after modification by surfactant adsorption. The graph describes the titration curve for same raspberry-like particles with two different smoothening silica layer thicknesses. The identical value of the isoelectric point for the two native raspberries indicates an identical surface chemistry[1]. The two curves fully overlap after surfactant adsorption.

Supplementary Note 5: Additional details of AFM roughness analysis

The surfaces of the scanned particles can be characterized by multiple descriptors, but since the surface topography of all fabricated rough particles is comparable, they were all described by a single parameter. In other words, all roughness features consist of spherical asperities and the differences between the different particle batches lies in the height, curvature and density of the spherical asperities, which are tuned by choosing the size and density of the silica berries and the thickness of the smoothening silica layer. We therefore chose to employ the root-mean-square roughness (*RMS* roughness) as the most appropriate parameter to describe and compare the surface topography of the different raspberry-like particles. The *RMS* roughness is defined as the root-mean-square of the surface vertical oscillations in the direction normal to the substrate and can be calculated as follows:

$$RMS = \sqrt{\frac{1}{n}\sum_{i=1}^{n} z_i^2} \qquad (1)$$

where z_i denotes the distance measured in the normal direction from the mean surface to the i-th data point; n is the total number of data points.

The *RMS* roughness is quite sensitive to the shape (in terms of slope and height of the protrusions), as well as to the area density of the asperities. To decouple the underlying particle curvature from the topographical signal of

interest, a custom-written algorithm based on a least-squares approach was used to find the unique sphere that closely encompasses the scanned particle (Supplementary Figure 3). Both the position of the particle center and its radius are determined as adjustable parameters. Most importantly, the roughness is calculated in the direction normal to the fitted sphere and not in the vertical direction of the AFM scanner.

In addition to the *RMS* roughness, the spatial distribution of the asperities was also extracted. To achieve this goal, the asperities' centers of mass were detected and the peak-to-peak distances were calculated using a Voronoi tessellation. Different asperity-to-asperity distances *D* were measured for the different batches of rough particles, depending on the used "berries" size. Asperities produced by adsorbing smaller particles naturally lay closer to each other, while asperities generated by bigger berries are farther apart (Supplementary Figure 4). To decouple the effect of the smoothening from the berries' size, the nearest inter-asperity distance was normalized by the berries' size. As shown in

Supplementary Figure 5, the smoothening is effectively leveling off the surfaces but the asperity-to-asperity distance remains in good approximation constant.

We additionally carried out simple numerical simulations to provide independent estimates of the *RMS* values and validate the experimental findings. Starting from a mathematically flat plane, we progressively added spherical asperities (Supplementary Figure 6a). As expected, by increasing the area fraction of the simulated asperities (with radius R and height H in agreement with the experimental values), the *RMS* roughness increases while the asperity-to-asperity distance decreases (Supplementary Figure 6b). For comparable nearest asperity distances *D*, the measured and simulated *RMS* values are in good agreement, confirming that our experimental description of the surface topography is reliable and robust (Supplementary Table 3). The smallest berries have a nominal diameter of 12 nm, but after coating with a silica layer their height and radius measured by AFM increased to 24 nm.

Thus, the smallest input radius and height for the simulations were set to 12 nm.

Supplementary Note 6: Capillary multipole deformations and their decay with the radial distance d

The profile of the interfacial deformation around a given particle can be compared to the predictions of the theory of capillarity. The theory[2, 3, 4] predicts that, in the case of negligible gravitational effects, the quadrupolar term is the leading term in the multipole expansion, describing the shape of the interface around a single particle with an undulated contact line. The amplitude of the quadrupolar deformation is predicted to decay as d^{-2} in the far-field, where d is the distance from the particle contour. The higher-order multipole deformations decay faster with the distance d and are thus neglected. In order to verify these theoretical predictions, which have never been directly tested for spherical microparticles with model rough surfaces, we fitted the interface profile at a fixed central angle, i.e. corresponding to a height maximum, with the expression

$$\mathrm{h(r)} = \mathrm{A_0} + \mathrm{A_2} \times \left(\frac{\mathrm{r_c}}{\mathrm{d}}\right)^2 \tag{2}$$

where r_c is the particle cross-sectional radius at the interface, d is the radial distance from the center of the contact line and A_i (i =0,2) are fitting parameters. A_0 takes into account the existence of a residual offset in the AFM images. A_2, i.e. the amplitude of the quadrupolar term, is a constant, since the interface profile is measured along a fixed central angle. The weight of the experimental data were scaled as $\sqrt{d}$ to ensure that the far-field deformation is correctly captured. The smooth particles with uniform surface chemistry do not deform the interface (Supplementary Figure 8a). In contrast, the rough particles (Supplementary Figure 8b) impart interfacial deformations, which can be accurately fitted with Supplementary Equation 2. This confirms the quadrupolar nature of the interfacial deformation, as predicted by the theory of capillarity.

Supplementary Note 7: Pinning energies

When a spherical particle with radius *r* adsorbs to a fluid-fluid interface, coming from the bulk phase where its surface energy is the lowest, i.e. water for hydrophilic particles or oil for hydrophobic particles, the adsorption energy is defined, neglecting line tension contributions, as[5]

$$\Delta F = -\gamma_{12}\pi r^2 \cdot (1 - |\cos(\theta)|)^2 \tag{3}$$

where the equilibrium contact angle θ is defined through Young's equation[6] $\cos(\theta) = (\gamma_{p2} - \gamma_{p1})/\gamma_{12}$. Supplementary Equation 3 is valid for $|\gamma_{p2} - \gamma_{p1}|/\gamma_{12} < 1$.

As reported by Nonomura and coworkers[7], roughness enlarges the surface area of the particles and consequently increases the adsorption energy. The increase in roughness is expressed mathematically by the surface area magnification factor α, which modifies Supplementary Equation 3 as follows

$$\Delta F_{rough} = -\gamma_{12}\pi r^2 \cdot (1 - \alpha \cdot |\cos(\theta)|)^2 \tag{4}$$

In Supplementary Equation 4, θ is again the contact angle for a smooth particle with the same surface chemistry.

When the rough particle is in a metastable position at the interface, its contact angle is θ_{eff} and its immersion depth z_{eff} is as follows:

$$z_{eff} = (1 + \cos\theta_{eff})r \tag{5}$$

The energy difference ΔF between the free energies of the particle at the metastable and equilibrium position at the interface is given by:

$$\begin{aligned} &\Delta F = F(z_{eff}) - F(z_{min}) = \\ &\pi r^2 \gamma_{12}\left\{(1+\cos\theta_{eff})^2 - 2(1+\cos\theta_{eff})(1+\alpha\cos\theta_0) + (1+\alpha\cos\theta_0)^2\right\} = \\ &\pi r^2 \gamma_{12}\left\{(1+\cos\theta_{eff}) - (1+\alpha\cos\theta_0)\right\}^2 = \pi r^2 \gamma_{12}(\cos\theta_{eff} - \alpha\cos\theta_0)^2 \end{aligned} \tag{6}$$

The excess area due to surface roughness was estimated from the AFM scans by normalizing the measured surface area of a rough particle by the corresponding underlying area of a smooth sphere. The data reported in Figure 2 of the main article show that rough particles are trapped in positions corresponding to contact angles that can be markedly different from the respective contact angle for a smooth particle with the same surface chemistry. Considering the contact angle of the smooth particles as the

condition of thermodynamic equilibrium and using Supplementary Equation 5, we can estimate how far from z_{min} are the trapped rough particles for the measured values of contact angles (θ_{eff}) and surface area magnification factor α. Surface tension values of 20 mN m^{-1} and 50 mN m^{-1} were selected for w/o interfaces with and without surfactants, respectively. If Wenzel's equation holds, ΔF is identically equal to 0. In practice, ΔF could differ from 0. However, to calculate ΔF accurately, one should know θ_0, θ_{eff} and α very precisely. Otherwise, the small errors of θ_0, θ_{eff} and α can be magnified by the large prefactor $\pi r^2\gamma_{12}$. In Supplementary Figure 9 are plotted the potential wells for the different roughness classes normalised by the equilibrium adsorption energy as

$$\frac{\Delta F}{\Delta F_{min}} = \frac{(\cos\theta_{eff} - \alpha\cos\theta_0)^2}{(1-\alpha|\cos\theta_0|)^2} \tag{7}$$

Supplementary Note 8: Dewetting of a single asperity (berry)

To assess whether thermal fluctuations can cause contact-line motion and ageing of contact angles[8], it is important to calculate the energy required for the contact line to move over a single asperity.

The free energy change for dewetting of a single asperity (berry) Δf_B is:

$$\Delta f_B = 2\pi r_B h\gamma_{12}\cos\theta_0\,, \tag{8}$$

where r_B is the radius of the berry; h is the height of the berry; and θ_0 is the contact angle of the smooth macroscopic material.

The free energy change for dewetting of the area below a single berry Δf_{Ref} is:

$$\Delta f_{Ref} = \pi(2r_B - h)h\gamma_{12}\cos\theta_0, \tag{9}$$

The free energy increase per berry Δf is thus given by:

$$\Delta f = \Delta f_B - \Delta f_{Ref} = \pi\left(2r_B h - 2r_B h + h^2\right)\gamma_{12}\cos\theta_0 = \pi h^2\gamma_{12}\cos\theta_0\,. \tag{10}$$

Δf depends on the berry height h, but not on its radius r_B.

From Supplementary Equation 10, for the smallest berries $h \approx 12$ nm and $\theta_0 \approx 70°$, $\Delta f \approx 2000\ k_BT$, way larger than thermal energy. These calculations imply that the metastable positions assumed by the rough particles upon adsorption are long-lived and negligible relaxation is to be expected, even in the presence of inputs of mechanical energy, as in emulsification processes.

Supplementary Note 9: Aggregation vs sedimentation for the unmodified 6 μm raspberry-like particles in decane

The sedimentation velocity of a single particle can be estimated from a simple force balance between the buoyancy force and viscous drag. The characteristic time scale for sedimentation is $\tau_s = \frac{r}{v_{s,0}} = \frac{9\eta_m}{2r\Delta\rho g}$, where η_m is the medium viscosity, r is the particle radius, g is the gravitational acceleration and $\Delta\rho$ is the density mismatch between the particle and the medium.

The characteristic time for Smoluchowski-like aggregation is $\tau_{agg} = \frac{3\eta_m}{4k_B T n}$, where *n* is the number of single particles per unit volume. At the typical particle concentrations (10^{14} particles per m^3) used for the preparation of FreSCa-samples, $\frac{\tau_s}{\tau_{agg}} = \frac{6k_B T n}{\Delta\rho g r} \sim 7\times10^{-5}$. Since $\tau_s \ll \tau_{agg}$, the silica particles with diameter of 6 μm sediment and reach the interface before having the possibility to aggregate.

Supplementary References

1. San-Miguel A, Behrens SH. Influence of Nanoscale Particle Roughness on the Stability of Pickering Emulsions. *Langmuir* **28**, 12038-12043 (2012).

2. Danov KD, Kralchevsky PA. Capillary forces between particles at a liquid interface: General theoretical approach and interactions between capillary multipoles. *Adv Colloid Interface Sci* **154**, 91-103 (2010).

3. Kralchevsky PA, Denkov ND, Danov KD. Particles with an Undulated Contact Line at a Fluid Interface: Interaction between Capillary Quadrupoles and Rheology of Particulate Monolayers. *Langmuir* **17**, 7694-7705 (2001).

4. Stamou D, Duschl C, Johannsmann D. Long-range attraction between colloidal spheres at the air-water interface: The consequence of an irregular meniscus. *Phys Rev E* **62**, 5263-5272 (2000).

5. Pieranski P. Two-Dimensional Interfacial Colloidal Crystals. *Phys Rev Lett* **45**, 569-572 (1980).

6. Young T. An Essay on the Cohesion of Fluids. *Philos Trans R Soc London* **95**, 65-87 (1805).

7. Nonomura Y, Komura S, Tsujii K. Adsorption of Microstructured Particles at Liquid–Liquid Interfaces. *J Phys Chem B* **110**, 13124-13129 (2006).

8. Kaz DM, McGorty R, Mani M, Brenner MP, Manoharan VN. Physical ageing of the contact line on colloidal particles at liquid interfaces. *Nat Mater* **11**, 138-142 (2012).
